# Сахарный диабет и опухоли женской репродуктивной системы

**DOI:** 10.14341/probl13282

**Published:** 2023-06-30

**Authors:** К. И. Шарафутдинова, В. С. Шляпина, А. И. Баева, А. A. Тимуршин, И. Э. Сабанаева, А. Г. Накиева, М. Ф. Калашникова, М. Н. Хабибов

**Affiliations:** Первый Московский государственный медицинский университет имени И.М. Сеченова (Сеченовский Университет); Российский национальный исследовательский медицинский университет имени Н.И. Пирогова; Российский национальный исследовательский медицинский университет имени Н.И. Пирогова; Башкирский государственный медицинский университет; Башкирский государственный медицинский университет; Башкирский государственный медицинский университет; Первый Московский государственный медицинский университет имени И.М. Сеченова (Сеченовский Университет); Первый Московский государственный медицинский университет имени И.М. Сеченова (Сеченовский Университет)

**Keywords:** сахарный диабет, опухоли эндометрия, опухоли яичников, сигнальные пути, белки новообразований

## Abstract

В статье обсуждаются различные патофизиологические состояния и процессы, приводящие к развитию опухолевых заболеваний при сахарном диабете. К ним относятся ожирение, гипергликемия, гиперинсулинемия, воспаление, а также окислительный стресс. Приводятся данные эпидемиологических исследований, в которых было установлено, что сахарный диабет (как 1-го, так и 2-го типа) повышает относительный риск развития опухолей женской репродуктивной системы, таких как рак яичников и рак эндометрия, тогда как для рака шейки матки, рака влагалища и рака вульвы такая взаимосвязь не была четко выявлена.

## Abstract (en)

The article discusses various pathophysiological conditions and processes that lead to the development of tumors in diabetes mellitus. These include obesity, hyperglycemia, hyperinsulinemia, inflammation, and oxidative stress. The data of epidemiological studies are given, in which it was found that diabetes mellitus (both type 1 and type 2) increases the risk of developing the female reproductive system tumors, such as ovarian cancer, endometrial cancer, while for cervical cancer, vaginal cancer and vulvar cancer, such a relationship has not been clearly identified.

## ВВЕДЕНИЕ

Предположение о том, что между сахарным диабетом (СД) и злокачественными новообразованиями существует взаимосвязь, было высказано еще более 100 лет назад [1]. Риск развития онкологических заболеваний повышается при СД как 1 типа (СД1), так и 2 типа (СД2) [2].Среди всех причин смерти пациентов с диабетом онкологические заболевания занимают второе место после сердечно-сосудистых заболеваний [3]. Также следует отметить, что примерно 8–18% больных раком страдают диабетом [4]. Проведенные мета-анализы и крупные популяционные исследования показали, что диабет связан с повышенным риском летальности при онкологических заболеваниях [5]. Однако основные механизмы связи между различными типами диабета и онкологическими заболеваниями (в особенности опухолями женской репродуктивной системы) подробно не обобщены. В данном обзоре производится анализ данной взаимосвязи, а также описываются механизмы ее возникновения.

## МАТЕРИАЛЫ И МЕТОДЫ

Настоящий обзор литературы проведен с целью критической оценки собранного материала. Авторами был выполнен электронный поиск публикаций в базах данных PubMed, Web of Science, ScienceDirect, Scopus. Условиями поиска было наличие слов «diabetes mellitus», «gynecologic cancers», «ovarian cancer», «endometrial cancer», «cervical cancer», «vaginal cancer» и «vulvar cancer». Авторы независимо друг от друга проанализировали те статьи, названия и аннотации которых были релевантны условиям поиска. Разногласия между авторами относительно приемлемости разрешали путем консенсуса. В обзор включены исследования, опубликованные преимущественно за последние 15 лет. Анализу подвергали полные тексты статей и их аннотации.

## ЭПИДЕМИОЛОГИЧЕСКИЕ АСПЕКТЫ

Число людей с СД продолжает возрастать во всем мире, что в значительной степени увеличивает нагрузку на систему здравоохранения. По последним данным, в 2021 г. распространенность диабета обоих типов в мире среди взрослого населения составила 537 млн человек, а к 2045 г. ожидается ее рост до 783 млн человек [6]. СД является достаточно распространенным заболеванием среди онкологических больных, при этом являясь доказанным фактором риска для некоторых солидных злокачественных новообразований, таких как рак поджелудочной железы, печени, толстой кишки, почки, мочевого пузыря и молочной железы [7].

Эпидемиологические исследования показали, что у людей с СД более часто встречается рак женской репродуктивной системы, включая рак эндометрия и яичников (РЯ). Один из метаанализов показал статистически значимую связь СД2 с риском развития рака эндометрия (общий относительный риск (ОР) 2,10; 95% доверительный интервал (ДИ) 1,75–2,53). Также в этом мета-анализе говорится и о связи СД1 и рака эндометрия (общий ОР 3,15; 95% ДИ 1,07–9,29). В исследовании Liu X. и соавт. приводятся данные, демонстрирующие, что пациенты с СД2 имеют повышенный риск развития уже упоминавшихся выше видов рака (толстой кишки, печени, поджелудочной железы, почек), в том числе и эндометрия (рис. 1). Авторы статьи предполагают, что повышенный риск развития онкологических заболеваний у пациентов с СД по сравнению с их родственниками связан с различными тяжелыми метаболическими нарушениями [8].

**Figure:**
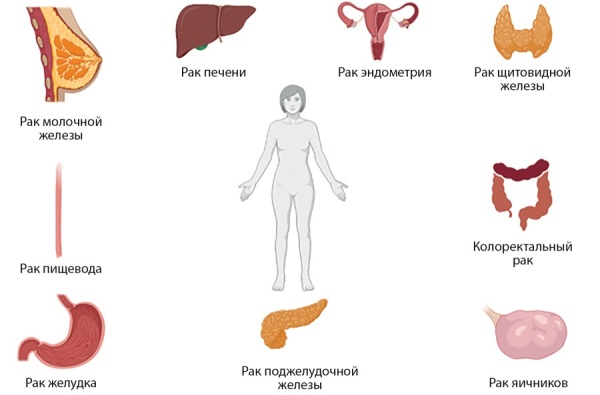
Рисунок 1. Наиболее распространенные онкологические заболевания при сахарном диабете.Figure 1. The most common cancers in diabetes mellitus

Следует отметить, что во многих исследованиях приводятся данные в пользу того, что заболеваемость раком яичников, пищевода, эндометрия, вульвы и влагалища, щитовидной железы выше среди женщин с СД1. С другой стороны, пациентки с СД1 имеют более низкий риск развития рака молочной железы, меланомы и лимфомы Ходжкина [9].

## ПАТОФИЗИОЛОГИЧЕСКИЕ СОСТОЯНИЯ И ПРОЦЕССЫ, ПРИВОДЯЩИЕ К РАЗВИТИЮ ОНКОЛОГИЧЕСКИХ ЗАБОЛЕВАНИЙ ПРИ САХАРНОМ ДИАБЕТЕ

## Гипергликемия

Эпидемиологические данные показали, что гипергликемия непосредственно связана с повышенным риском колоректального рака, рака печени, желудка, легких и поджелудочной железы [10].

Клетки большинства типов злокачественных новообразований преимущественно экспрессируют глюкозный транспортер типа 1, который имеет высокое сродство к глюкозе. Также стоит упомянуть и эффект Варбурга, при котором в опухолевых клетках глюкоза идет по энергетически неэффективному пути гликолиза с высоким уровнем продукции лактата. Повышенный гликолиз в клетках опухолей обеспечивает материалы, необходимые для синтеза нуклеотидов, аминокислот и липидов [11].

Гипергликемия стимулирует выработку конечных продуктов гликозилирования (КПГ). КПГ взаимодействуют со специфическим рецептором КПГ, что приводит к активации сигнального пути NF-kB (ядерный фактор κB) и образованию активных форм кислорода (АФК) в клетках, что тем самым ускоряет окислительный стресс и приводит к активации провоспалительных сигнальных путей [10]. Было продемонстрировано, что КПГ и активация нижележащих путей способствуют опухолевой трансформации эпителиальных клеток [12]. Показано, например, что гипергликемия стимулирует пролиферацию раковых клеток поджелудочной железы посредством индукции экспрессии эпителиального фактора роста (EGF) и трансактивации его рецептора (EGFR) [13]. EGF и его рецептор, в свою очередь, играют важную роль в патогенезе опухолей женской репродуктивной системы, например, рака яичника и рака шейки матки (РШМ) [14]. Кроме того, гипергликемия ответственна за повреждение ДНК, которое является первой стадией онкогенеза [15]. Для рака эндометрия было высказано предположение, что при предраковых состояниях или уже имеющемся опухолевом заболевании клетки эндометрия, а также клетки микроокружения могут использовать избыточную глюкозу для стимулирования анаболических путей и прогрессирования заболевания. Генетические особенности рака эндометрия указывают на то, что большинство этих новообразований уже имеют предрасположенность к повышенному метаболизму глюкозы за счет потери экспрессии PTEN (phosphatase and tensin homolog deleted on chromosome 10) и/или активации пути PI3K/Akt (фосфоинозитид-3-киназа/протеинкиназа B) посредством других механизмов [16].

## Гиперинсулинемия

При СД2 инсулинорезистентность приводит к развитию компенсаторной гиперинсулинемии. Высокая концентрация инсулина в крови играет одну из ключевых ролей в патогенезе злокачественных новообразований, что было продемонстрировано в многочисленных исследованиях как in vitro, так и in vivo [17].

На клеточной культуре рака эндометрия было установлено, что, с одной стороны, гиперинсулинемия приводит к замедлению процесса мезенхимально-эпителиального перехода (как следствию, метастазирования и инвазии опухоли) посредством ингибирования экспрессии COL1A1 (гена, кодирующего α1-цепь коллагена I типа), IRS2 (субстрата инсулинового рецептора-1), однако, с другой стороны, повышает уровень экспрессии AMFR (Autocrine Motility Factor Receptor), FAF1 (FAS-ассоциированного фактора 1), MPP3 (Membrane Palmitoylated Protein 3), PIP2 (фосфатидилинозитол-4,5-дифосфата), VEGFA (фактора роста эндотелия сосудов), а также генов других белков, способствующих развитию и прогрессированию опухоли [18].

Предполагается, что метаболические эффекты инсулина, к которым относится транспорт глюкозы в клетку, опосредован активацией сигнального пути PI3K/Akt, тогда как митогенный эффект оказывается на клетки посредством Ras/Raf/MAPK (Rat sarcoma virus/RAF proto-oncogene serine/threonine-protein kinase/Mitogen-Activated Protein Kinase) сигнального пути, в данном пути нижележащими являются киназы MEK (Mitogen-Activated Kinase) и ERK (Extracellular signal-Regulated Kinase). Проинсулин и инсулин оказывают сходное стимулирующее действие на активацию MAPK, пролиферацию и миграцию клеток рака молочной железы [19].

Также необходимо отметить важность инсулиноподобных факторов роста (ИФР), участвующих в развитии злокачественных опухолей [20]. Гиперинсулинемия, сопровождающая периферическую инсулинорезистентность, приводит к снижению секреции гормона роста, в то время как общий уровень ИФР-1 остается относительно неизменным из-за повышенной чувствительности печени к гормону роста. Уровень ИФР-связывающего белка (IGFBP-1) снижается в связи с повышением уровня инсулина, что приводит к повышению биодоступности ИФР-1 [15]. Имеются и другие данные о нормальных или повышенных уровнях ИФР-1 у пациентов с ожирением [21]. ИФР способствуют росту и выживанию клеток, прогрессии клеточного цикла, подавляют апоптоз, усиливают ангиогенез и метастатическую активность при различных видах рака. Эти факторы напрямую регулируют клеточные функции, взаимодействуя со специфическими рецепторами клеточной поверхности и активируя внутриклеточные сигнальные пути [22].

## Ожирение

Широко известно, что большинство пациентов с предиабетом или СД2 имеют избыточный вес или ожирение [23]. При ожирении, по данным рабочей группы Международной ассоциации изучения рака, наблюдается повышенный риск развития 13 различных новообразований, включая рак эндометрия, пищевода, почек и поджелудочной железы, гепатоцеллюлярную карциному, рак кардиального отдела желудка, менингиому; множественную миелому, колоректальный рак, РМЖ в постменопаузе, рак яичников, желчного пузыря и щитовидной железы [24]. «Зонтичный» обзор систематических обзоров и 204 мета-анализов также выявил взаимосвязь между ожирением и развитием рака. Данная связь особенно была выражена для рака желудочно-кишечного тракта и рака женской репродуктивной системы [25]. Данные 8 наблюдательных исследований с участием 635 642 пациентов свидетельствуют о том, что бариатрическая хирургия связана со снижением риска развития всех видов рака (обобщенное отношение шансов (ОШ) 0,72; 95% ДИ 0,59–0,87), а также рака, связанного с ожирением (обобщенное ОШ 0,55; 95% ДИ 0,31–0,96), включая рак молочной железы (обобщенное ОШ 0,50; 95% ДИ 0,25–0,99) [26].

Также при ожирении жировая ткань продуцирует большое количество провоспалительных цитокинов и адипокинов, что приводит к повышению уровня окислительного стресса. Предполагается, что это приводит к развитию особого микроокружения опухоли, тем самым способствует росту опухолевых клеток, усиливает метастазирование, ангиогенез и нарушает функцию естественных клеток-киллеров и макрофагов [27]. Более подробно участие воспаления в развитии опухолей обсуждается ниже.

Кроме того, при ожирении сниженная секреция адипонектина и повышенная секреция лептина жировой тканью могут способствовать развитию рака. Адипонектин обладает противовоспалительными и антиангиогенными свойствами и может ингибировать рост рака. Некоторые опухолевые клетки экспрессируют рецепторы адипонектина, таким образом, адипонектин, связывая и активируя эти рецепторы, может запускать сигнальные пути в раковых клетках, а дефицит адипонектина исключает такое действие [28]. Лептин является митогенным, антиапоптотическим, проангиогенным и провоспалительным фактором [29]. Связанные с ожирением нарушения секреции адипокинов и цитокинов приводят к активации онкогенных сигнальных путей, таких как NF-kB, JAK2/STAT3 или PI3K/Akt [30]. NF-κB играет важную роль в модуляции ответа раковых клеток на повреждение ДНК [31]. Гиперинсулинемия, обычно наблюдаемая при ожирении, может повышать активность PI3K/Akt и влиять на функцию p53. Активирующие мутации p53 усиливают Akt и, в свою очередь, влияют на ответ раковых клеток на инсулин, что приводит к усилению пролиферации и миграции [32].

Большой массив данных свидетельствует о том, что гипергликемия является фактором, способствующим возникновению и прогрессированию эпителиального РЯ через ряд механизмов, включая гиперинсулинемию, усиление ангиогенеза, воспаления, повреждение ДНК. Предполагается, что гипергликемия оказывает важное влияние как на раковые клетки, так и на прогрессирование эпителиального рака яичников [33].

## Воспаление и окислительный стресс

Воспаление является одним из ключевых связующих элементов между СД и онкологическими заболеваниями. Хроническое слабовыраженное воспаление в гипертрофированной жировой ткани играет важную роль в патогенезе инсулинорезистентности, связанной с ожирением [34]. В свою очередь, хроническое воспаление связано с повышением уровня уже упоминавшихся окислительного стресса и АФК, которые имеют решающее значение для многих процессов в развитии рака [35]. Кроме того, из-за гипергликемии конечные продукты усиленного гликозилирования и их рецепторы приводят к развитию окислительного стресса и усилению воспаления, что способствует росту клеток, ангиогенезу и метастазированию [11].

В ходе различных исследований были выявлены повышенные уровни циркулирующих провоспалительных цитокинов IL-1 (интерлейкин-1), IL-6 (интерлейкин-6) и TNF-α (фактор некроза опухоли-альфа) у пациентов с СД [36]. TNF-α способствует опухолевому развитию за счет активации NF-kB и увеличения продукции АФК и активных форм азота, которые могут вызывать повреждение ДНК. Данный цитокин также стимулирует рост, пролиферацию, инвазию и метастазирование опухолевых клеток, а также ангиогенез [37].

Повышенные уровни IL-6 были обнаружены у пациентов с раком поджелудочной, молочной железы, В-клеточной лимфомой и миеломой [38]. IL-6 играет важную роль в стимулировании пролиферации опухолевых клеток и в ингибировании апоптоза посредством связывания рецептора IL-6 с gp130 (гликопротеин 130). Далее происходит активация сигнального пути JAK/STAT (янус-киназа/Signal Transducer and Activator of Transcription), а именно STAT1 и STAT3 [39], что приводит к прогрессированию опухоли. Подобно TNF-α, IL-6 стимулирует развитие опухоли, индуцируя преобразование нормальных эпителиальных клеток в раковые стволовые клетки [40].

Для IL-1 также было показано, что его уровень повышается при некоторых типах опухолей, таких как опухоли молочной железы, толстой кишки, головы и шеи, легких, поджелудочной железы и меланома. Кроме того, пациенты с высоким уровнем IL-1 обычно имеют неблагоприятный прогноз [41]. IL-1 вынуждает эпителиальные и предраковые клетки продуцировать молекулы, повреждающие ДНК, такие как АФК и оксид азота, что приводит к накоплению мутаций [42], что связано со способностью воспалительных цитокинов повышать активность AICDA (Activation-Induced Cytidine Deaminase). Последний вызывает геномную нестабильность и мутации при многих типах рака [43]. Схема, обобщающая все вышеназванные состояния и процессы, которые приводят к развитию опухолей при СД, приведена на рис. 2.

**Figure:**
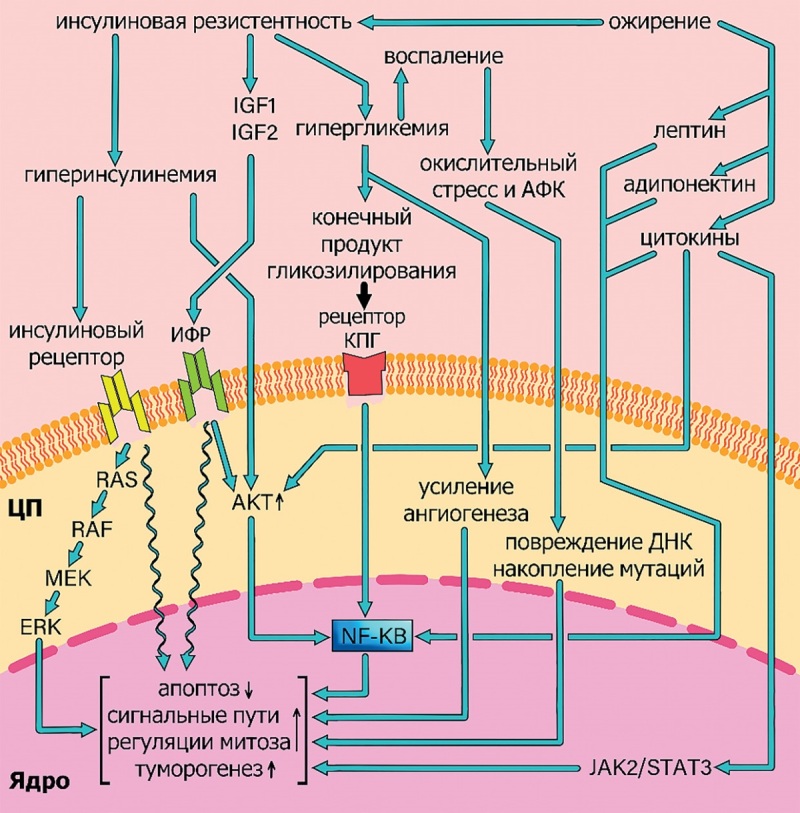
Рисунок 2. Основные механизмы возникновения опухолевых заболеваний при сахарном диабете.АФК — активные формы кислорода; КПГ — конечные продукты гликозилирования; ИФР — инсулиноподобные факторы роста; ЦП — цитоплазма; AKT — протеинкиназа B; ERK — Extracellular signal-Regulated Kinase; IGF — инсулиноподобные факторы роста; MEK — МАРК-Extracellular regulated Kinase; NF-KB — ядерный фактор κB; RAF — RAF proto-oncogene serine/threonine-protein kinase; RAS — Rat Sarcoma virus; JAK2/STAT3 — киназа Janus 2/Signal Transducer and Activator of Transcription 3).Figure 2. The main mechanisms of the onset of tumor diseases in diabetes mellitus

При сахарном диабете возникает инсулинорезистентность, которая приводит к гиперинсулинемии, повышению уровней инсулиноподобных факторов роста IGF1 и IGF2, а также гипергликемии. Гиперинсулинемия активирует каскад RAS с последующей активацией RAF, MEK и ERK, а также путь AKT с последующей активацией NF-KB, что вызывает канцерогенные эффекты, такие как снижение уровня апоптоза, активация сигнальных путей, ответственных за деление клетки. Гипергликемия усиливает ангиогенез, а также вызывает избыточную продукцию КПГ, которые активируют соответствующий рецептор и вызывают активацию пути NF-KB. Кроме того, гипергликемия усиливает воспалительный ответ, что связано с канцерогенными эффектами СД через окислительный стресс и повреждение ДНК. Ожирение также связано с канцерогенными эффектами через активацию путей AKT и JAK/STAT3, вызванных избыточной продукцией лептина, адипонектина и различных цитокинов, таких как IL-6, IL-1β и TNF-α.

## ОНКОЛОГИЧЕСКИЕ ЗАБОЛЕВАНИЯ ЖЕНСКОЙ РЕПРОДУКТИВНОЙ СИСТЕМЫ И САХАРНЫЙ ДИАБЕТ

## Рак эндометрия

Эпидемиологические данные свидетельствуют о тесной связи между раком эндометрия и ожирением, описаны многочисленные механизмы данной взаимосвязи [44]. Во многих других эпидемиологических исследованиях также было сообщено о связи между СД и риском развития рака эндометрия [45]. Фактически ожирение, гиперинсулинемия и инсулинорезистентность являются известными модифицируемыми факторами риска развития СД2 и, как было показано, факторами риска развития рака эндометрия [46].

В систематическом обзоре и мета-анализе обсервационных исследований по изучению связи между диабетом и риском развития рака эндометрия было показано, что суммарный относительный риск составил 1,89 (95% ДИ 1,46–2,45), а суммарный коэффициент частоты заболеваемости — 1,61 (95% ДИ 1,51–1,71) для рака эндометрия среди женщин с СД2 по сравнению с женщинами без СД2 [45]. Другой систематический обзор был посвящен сравнению выживаемости пациенток с раком эндометрия, у которых на момент постановки онкологического диагноза был СД, с теми, у кого не было СД. Результаты показали, что наличие СД связано с более низкой общей выживаемостью, но не с выживаемостью без заболевания, безрецидивной выживаемостью или выживаемостью без прогрессирования [47]

Кроме того, у женщин с диагностированной гиперплазией эндометрия СД является фактором риска развития рака эндометрия и поэтому может быть включен в прогностический алгоритм для стратификации риска [48].

Хотя влияние диабета на риск развития рака эндометрия может быть небольшим, учитывая высокую распространенность диабета и рака эндометрия, последствия для общественного здравоохранения являются значительными [49].

Lotfolah Saed и соавт. (2019) в метаанализе пытались оценить влияние диабета на риск заболеваемости раком эндометрия. В исследовании было показано, что риск развития рака эндометрия повышен у пациентов с СД на 72% процента [49], что согласуется с результатами предыдущего метаанализа, проведенного E. Friberg и соавт. [46]. Авторы подчеркивают, что данный риск может быть завышен вследствие нескольких искажающих факторов: малоподвижный образ жизни, гормональные нарушения и ожирение. Последний является одним из важнейших факторов развития диабета, приводящим к гормональному дисбалансу (у женщин с ожирением выше уровень эстрогенов за счет продукции эстрона), что предрасполагает к развитию рака эндометрия. Точные механизмы канцерогенеза эндометрия неясны, предполагается, что основная роль заключается в избыточном воздействии эстрогена на эндометрий, которое не компенсируется воздействием прогестерона, что приводит к повышенной митогенной активности клеток эндометрия. Также имеются данные, что высокие уровни инсулина, факторов роста, а также длительная инсулинотерапия могут способствовать развитию рака эндометрия [49]. Было отмечено, например, что у пациенток с имеющимся раком эндометрия при СД достоверно повышены уровни ИФР в опухолевой ткани [50].

В заключение следует отметить, что диабет, по-видимому, увеличивает риск рака эндометрия у женщин, что может быть полезным при разработке профилактики рака эндометрия для женщин, страдающих диабетом [49]. Существуют доказательства того, что диабет повышает риск развития рака эндометрия, и для предотвращения риска прогрессирования заболевания у женщин с диагностированной гиперплазией эндометрия может потребоваться гликемический контроль [48]. Необходимы дальнейшие исследования для определения точного механизма канцерогенного влияния диабета на рак эндометрия.

## Рак яичников

Связь РЯ и СД (а именно СД1) была изучена в популяционных исследованиях. Так, в мета-анализе китайских ученых, включающем 12 когортных исследований, ОР неспецифической смертности составил 1,44 (95% ДИ 1,16–1,79) без существенной статистической неоднородности. Относительный риск опухоль-специфической смертности также составил 1,44 (95% ДИ 1,08–1,93) со значительной неоднородностью [51].

Систематический обзор и мета-анализ обсервационных исследований показали, что у женщин с СД относительный риск развития РЯ на 19% выше, чем у женщин без СД [52]. В другом метаанализе также сообщалось о положительной связи между СД и РЯ с суммарным ОР 1,23 (95% ДИ: 1,12–1,36) [53].

Кроме того, диабет отрицательно влияет на онкологические исходы у пациентов при некоторых других видах рака, но его влияние на течение рака яичников плохо изучено [54]. В некоторых исследованиях также изучалось влияние диабета на выживаемость женщин с РЯ. Было показано, что диабет оказывает влияние на течение РЯ со снижением выживаемости без прогрессирования и общей выживаемости [54]. Другое исследование также показало, что женщины с СД имеют более низкую выживаемость при РЯ [55].

Таким образом, несмотря на то, что на настоящий момент получены доказательства влияния СД на риск развития РЯ, связь между этими двумя состояниями сложна и требует дальнейшего изучения [51].

## Рак шейки матки

В одном из метаанализов было показано, что СД является важным прогностическим фактором для РШМ, который связан со снижением как общей, так и безрецидивной выживаемости. Авторы статьи считают, что диабет может служить полезным показателем при прогностической оценке больных РШМ [56]. Однако в другом исследовании, где было изучено влияние СД на выживаемость пациентов с РШМ, не было отмечено существенной разницы в общей и безрецидивной выживаемости между пациентами с СД и без него, хотя в группе без СД наблюдалась тенденция к увеличению выживаемости. Исследования показали, что СД является отрицательным прогностическим фактором для других видов рака [57]. Так или иначе, не было установлено прямой связи между имеющимся СД и риском развития РШМ [58].

Также стоит отметить, что в настоящее время связь между СД и риском развития РШМ достаточно плохо изучена. Наиболее часто предполагаемыми механизмами, лежащими в основе данной взаимосвязи (как и для других новообразований, что обсуждалось выше), являются инсулинорезистентность, которая приводит к вторичной гиперинсулинемии, митогенное действие инсулина, оказываемое через рецептор ИФР, а также гипергликемия, усиливающая канцерогенез за счет индукции окислительного стресса [57]. Однако до сих пор неясно, может ли гиперинсулинемия способствовать развитию и прогрессированию именно рака шейки матки [59].

## Рак влагалища, вульвы

Важно отметить, что в настоящее время нет убедительных доказательств наличия прямой связи между СД и раком влагалища. Однако поскольку другие виды рака женской половой системы имеют сходные факторы риска с раком влагалища, вероятность такой связи существует для уточнения характера влияния СД на данный вид [60]. Необходимы дальнейшие исследования.

Для рака вульвы авторы выявили определенную корреляцию с СД2 [58], однако других опубликованных работ, оценивающих данную связь, нами не было найдено.

## ЗАКЛЮЧЕНИЕ

Опухоли женской репродуктивной системы по-прежнему остаются серьезной проблемой как для пациентов, так и системы здравоохранения. В статье проведен литературный анализ оценки влияния хронической гипергликемии, гиперинсулинемии, системного воспаления и окислительного стресса, а также изменения уровня половых гормонов, наблюдаемых при СД, на формирование опухолей женской репродуктивной системы. Учитывая тот факт, что онкологические заболевания являются второй причиной смерти пациентов с СД1 и СД2, приведенные в настоящем обзоре результаты исследований свидетельствуют в пользу целесообразности проведения скрининга онкологических заболеваний репродуктивной системы у женщин с СД, роль в этом также должна играть диспансеризация. Кроме того, необходимо принимать меры первичной профилактики у пациенток с СД, в частности снижать вес, строго контролировать течение заболевания. Следует также проявлять настороженность в отношении онкологических заболеваний при диагностике СД. Необходимы дальнейшие исследования для определения биологических механизмов и разработки эффективных мер по профилактике и лечению опухолей репродуктивной системы у женщин с сахарным диабетом.

## ДОПОЛНИТЕЛЬНАЯ ИНФОРМАЦИЯ

Источники финансирования. Работа выполнена по инициативе авторов без привлечения финансирования.

Конфликт интересов. Авторы заявляют об отсутствии конфликта интересов.

Участие авторов. Шарафутдинова К.И. — концепция и дизайн исследования, написание текста; Шляпина В.С. — сбор и обработка материала, редактирование; Баева А.И. — сбор и обработка материала, редактирование; Тимуршин А.А. — написание текста, редактирование; Сабанаева И.Э. — сбор и обработка материала, редактирование; Накиева А.Г. — концепция и дизайн исследования, сбор и обработка материала; Калашникова М.Ф.— обработка материала и редактирование текста; Хабибов М.Н. — концепция и дизайн исследования, написание текста. Все авторы внесли равный вклад в написание статьи, одобрили финальную версию статьи перед публикацией, выразили согласие нести ответственность за все аспекты работы, подразумевающую надлежащее изучение и решение вопросов, связанных с точностью или добросовестностью любой части работы.
